# Development and validation of a QuEChERS-LC-MS/MS method for determination of multiple mycotoxins in maize and sorghum from Botswana

**DOI:** 10.3389/ffunb.2023.1141427

**Published:** 2023-08-03

**Authors:** Mesha Mbisana, Tshepho Rebagamang, Dikabo Mogopodi, Inonge Chibua

**Affiliations:** ^1^ Laboratory of Analytical Chemistry, Department of Chemistry, Faculty of Science, University of Botswana, Gaborone, Botswana; ^2^ Residues, Botswana National Veterinary Laboratory, Gaborone, Botswana

**Keywords:** multiple mycotoxins, food safety, maize, sorghum, QuEChERS-LC-MS/MS, method development

## Abstract

Climatic conditions such as drought, high temperatures, and pre-harvest rainfalls promote the occurrence of mycotoxins in grains. Contamination of staple food sources such as maize and sorghum means that many populations are at risk of being poisoned by mycotoxins. Hence the need for sensitive methods for their simultaneous analysis. Herein, a quick, easy, cheap, effective, rugged, and safe liquid chromatography tandem mass spectrometry (QuEChERS-LC-MS/MS) method for the simultaneous determination of ten mycotoxins in maize and sorghum is presented. The QuEChERS extraction procedure was optimized to maximize extraction recovery and minimize matrix effects while using relatively small quantities of organic solvents and acids. This method was validated according to Commission Implementing Regulation (EU) 2021/808, Commission Regulation (EC) No 1881/2006, and Regulation (EC) no. 401/2006. The developed method met the specified requirements. Recoveries of 80.77% to 109.83% and CVs below 15% were obtained. The correlation coefficient values (R^2^) were all above 0.98, and low limits of quantification ranging from 0.53 to 89.28 µg/Kg were recorded. The method was applied to 10 maize and 10 sorghum samples collected from markets in Botswana. Half of the samples had detectable mycotoxins, Aflatoxins, Fumonisins, T2-toxin, HT2-toxin, and Zearalenone. Two maize samples had levels of aflatoxin B_1_ above the maximum permitted level (2.55, 4.07 µg/Kg). These findings point to the necessity of more stringent monitoring of mycotoxins, particularly AFB1 in maize, as well as the value of regular assessment using LC-MS/MS.

## 1 Introduction

Mycotoxins are toxic metabolites produced by fungi. The fungi grow on food commodities such as cereals, coffee, fruits, nuts, oilseeds, and spices when there are favorable conditions (Awuchi et al., 2021). Currently, over 400 mycotoxins have been recorded, and 25% of food has been shown to be contaminated. (Tola and Kebede, 2016; Escrivá et al., 2017); however, only a few are of concern from a food safety perspective: Aflatoxins (AFs), Fumonisins (FBs), Ochratoxin A (OTA), Zearalenone (ZEA), Egort alkoids (EAs), Enniantis (ENs), Patulin (PAT), and Trichothecenes (Barac, 2018). Mycotoxins can affect human and animal health in different ways. Hepatoxicity (liver damage), genotoxicity (damage to genetic information), immunosuppression, and teratogenicity (damage to an embryo or fetus) are the most common.

The detrimental health consequences of mycotoxins have led to the establishment of maximum allowable levels in food and feed by several regulatory authorities. The European Food Safety Authority regulates mycotoxins of concern in the European Union, including AFs, FBs, OTA, trichothecenes, and ZEA (Lawley, 2013; EC, 2019). The same regulations have been adopted in Botswana, and they are implemented by the Botswana National Veterinary Laboratory (BNVL).

The occurrence of mycotoxins in Botswana has been scarcely studied. The latest study involved the identification of mycotoxigenic fungi in maize, millet, sorghum and peas using the polymerase chain reaction (Masitha et al., 2019). Various fungi species such as *Aspergillus (flavus, niger), Fusarium (proliferatum, fujikuroi)* and *Alternaria* were found in 40%, 37%, 27%, 10%, and 4% of millet, yellow maize, white maize, cowpeas, and red sorghum, respectively. This indicated possible exposure of humans and animals in Botswana to mycotoxins. However, the earlier findings by Mupunga (2013), who studied the natural contamination of peanuts, peanut butter, and sorghum with AFs in Botswana, reported that no food commodities in Botswana were contaminated. Numerous studies have been conducted on food and feed contamination in Africa. Chauhan summarized foods that are susceptible to aflatoxin contamination, as adapted from several sources. The study revealed that staple foods in most countries are contaminated with AFs, which shows that mycotoxins further jeopardize the United Nations sustainable development goals to end hunger and establish good health and nutrition (Chauhan, 2017).

Losses resulting from mycotoxin contamination of food are of serious economic impact as they are trade-related because produce often doesn’t meet the international trade standards. Consequently, the food is sold to consumers with low incomes, which results in lower profits (Imade et al., 2021). This is a common occurrence in most African counties (Marechera and Ndwiga, 2015). Considering these economic impacts and the fact that mycotoxins can be harmful even at low doses, it is critical to develop sensitive, effective, and rapid analytical methods for their determination, especially those that can simultaneously analyze multiple mycotoxins in food. Extraction, isolation, and cleanup are common steps in determining mycotoxins, followed by detection, qualification, and quantification.

The QuEChERS method, initially developed for pesticides (Anastassiades et al., 2003), has been applied to mycotoxins due to its benefits, as implied by its name, and has yielded high-quality results (Sirhan et al., 2014; Jo et al., 2021; Pantano et al., 2021). In terms of quantification, LC-MS/MS has proven to be the most effective method for measuring mycotoxins because it combines the separating power of high performance liquid chromatography with the detection power of triple quadrupole mass spectrometry (Pascale et al., 2019). The mass spectrometer enables simultaneous detection of many metabolites due to its unique principle, which offers unequalled sensitivity, detection limits, and speed (Hoffmann and Stroobant, 2007). As many as 33 different mycotoxins have been simultaneously determined using mass spectrometric detection (Spanjer et al., 2008). As a result of the wide variety of possible pairings between the stationary phase and the mobile phase, a separation may be designed to accommodate a wide variety of intricate problems. Several studies have reported the LC-MS/MS method in the determination of multiple mycotoxins, such as 13 different mycotoxins in maize and beans (Jo et al., 2021), 12 mycotoxins in cereal products and spices (Pantano et al., 2021), and 33 mycotoxins in peanuts, maize, raisins, and wheat (Spanjer et al., 2008). However, these studies do not include comprehensive validation based on appropriate regulations. **Table 1** shows publications where LC-MS/MS was used to determine mycotoxins in various food matrices from 2018–2021. The standard technique for evaluating ZEA levels in edible vegetable oils and T2/HT2 mycotoxins in cereals and cereal products was recently released by the European Committee for Standardization (CEN) (CEN, 2017a; CEN, 2017b).

**Table 1:** Studies in which LC-MS/MS was used to determine multiple mycotoxins in various food matrices (2018-2021).

No. of Mycotoxins determined	Year of publication	Sample	Extraction solution	Clean up	LOD	LOQ	Reference
**28**	2018	Human breast milk	MeCN with 1% formic acid	LLE and SPE	0.004-1.4 ng/ml	0.009-2.9 ng/ml	(Braun et al., 2018)
**5**	2018	Corn flour	MeCN: H_2_O: FA (79: 20: 1)	dSPE	0.6 – 25 ng/g	2 – 75 ng/g	(Amirahmadi et al., 2018)
**16**	2019	Cereals	MeCN: H2O (80:20)	–	–	0.001-0.2 mg/kg	(Kresse et al., 2019)
**34**	2020	Human breast milk	MeCN with 0.1% FA	SPE	0.1 – 300 ng/L	0.2 – 600 ng/L	(Braun et al., 2020)
**9**	2020	Grain legumes	H_2_O and MeCN with 0.1% FA	–	0.1 – 6.4 µg/Kg	0.3 – 21.3 µg/Kg	(Kunz et al., 2020)
**22**	2021	Cereals and oil seeds	MeCN	–	–	0.22 – 32.64 µg/Kg	(González-Jartín et al., 2021)
**2**	2021	Soil	MeCN: H_2_O: AcOH (79: 20: 1)	–	–	0.5 – 1 ng/g	(Kappenberg and Juraschek, 2021)
**13**	2021	Rice bran	MeOH: H_2_O (80:20) and CHCl_3_	–	0.5 – 50 ng/g	1.5 – 150 ng/g	(Salim et al., 2021)
**12**	2021	Cereals and spices	MeCN: FA (80:20)	dSPE	–	–	(Pantano et al., 2021)
**13**	2021	Feedstuffs	H_2_O: FA (90:10) and MeCN	dSPE	–	–	(Jo et al., 2021)

Results of literature search for studies that employed LC-MS/MS for determination of multiple mycotoxins in various matrices. Year of publication, matrix, extraction solvents, clean up techniques, LODs and LOQs are specified.

The purpose of this study was to develop an easy and sensitive approach for the identification of several mycotoxins using a simplified QuEChERS extraction method and LC-MS/MS detection. Using the one factor at a time (OFAT) approach, five factors affecting the QuEChERS extraction efficiency were optimized. The method was then validated according to Commission Implementing Regulation (EU) 2021/808 of 22 March 2021 and Regulation (EC) no. 401/2006. EC and applied to determine the occurrence of ten mycotoxins in maize and sorghum samples collected from markets in Botswana. To the best of our knowledge, this is the first report on the occurrence of multiple mycotoxins in maize and sorghum from Botswana.

## 2 Materials and methods

### 2.1 Chemicals and reagents

Mycotoxin standards (AFB_1_, AFB_2_, AFG_1_, AFG_2_, OTA, HT2-Toxin, T2-Toxin, FB_1_ and FB_2_) were purchased from Trigology Analytical Laboratory, Inc (USA), and ZEA was supplied by Sigma Aldrich (Austria). Lichrosolv chemicals and reagents were purchased from Merck, Supelco (Germany); ultrapure water (H_2_O) for chromatography (LC-MS grade), methanol (MeOH) (hyper-grade for LC-MS), and acetonitrile (MeCN) (gradient grade for liquid chromatography). Formic acid (FA) (ACS, >98%) and Dimethyl Sulfoxide (DMSO) (> 99.8%) were purchased from Carl Roth (Germany). Sodium chloride (NaCl) analytical reagent and magnesium sulphate (MgSO_4_) were obtained from Rochelle Chemicals (South Africa). Anhydrous MgSO_4_ was obtained from Parks Scientific.

### 2.2 Sample collection and treatment

Blank maize sample was purchased from Choppies supermarket in Gaborone, and a blank sorghum sample was provided by the Botswana National Veterinary Laboratory. Twenty samples were collected from Francistown and Gaborone city markets. Ten maize and ten sorghum samples (about 1 kg each) were finely blended using a laboratory blender and stored in a tightly closed plastic honey jar. All samples were kept in the freezer at -20°C until analysis.

### 2.3 Preparation of multi-mycotoxins standard solution

To obtain a standard solution of AFB_1_, AFB_2_, AFG_1_, AFG_2_ (10 µg/L), FB_1_, FB_2_, T2-toxin (250 µg/L), HT2-toxin, ZEA (100 µg/L), and OTA (40 µg/L), 200 µL of each AF standard (1.25 µg/mL), 1250 µL of FB_1_, FB_2_ and T2-toxin standards (5.00 µg/mL), 500 µL of HT2-toxin, ZEA standards (5.00 µg/mL) and 2000 µL of OTA standard (0.50 µg/mL) were all pipetted into a 25 mL volumetric flask, which was then filled to the mark using MeOH. The standard solutions were kept in amber screw-cap bottles at -20°C when not in use.

### 2.4 Preparation of maize and sorghum sample extracts via the modified QuEChERS procedure

In a 50 mL centrifuge tube, 2.0 ± 0.1g of finely ground sample was extracted with 8 mL MeCN/H2O (80:20 v/v) containing 0.1% FA. The mixture was homogenized for 30 seconds with an Ultra-Torrax T25 (Optolabor, Durban) probe homogenizer and then left to shake for 60 minutes on a PSU-20i electronic shaker for maximum extraction. After shaking was complete, 1 g of NaCl and/or 4 g MgSO_4_ was added to the mixture for extract cleaning and clear face separation, followed by vortex for 1 minute on a Heldolph multireax. The sample was centrifuged using a Thermo Scientific Haraeus Megafuge 16R Centrifuge (IAEA, Austria) for 10 minutes at 5000 rpm, 4°C. Thereafter, 5 mL of supernatant was collected into a glass tube and preconcentrated to 500 µL over a gentle stream of nitrogen gas (40°C) using a turbovap then reconstituted with 500 µL MeOH/H2O (50:50 v/v). The sample was then filtered through a 0.45 µm syringe filter and ready for injection into LC-MS/MS.

### 2.5 LC-MS/MS parameters and analysis

The analysis was performed using an ExionLC™ LC coupled with a binary gradient AD pump, AD autosampler, AD column oven and linear ion trap quadrupole (QTRAP 6500^+^) mass spectrometer with an electron spray ionization (ESI) (IonDrive TM Turbo V source) manufactured by AB Sciex Instruments (USA). Separation employed a Zorbax Eclipse Plus C18 (2.1 x 150 mm, 5 µm, Agilent, USA) column. Mobile phase A was composed of H_2_O + 0.1% FA while mobile phase B was composed of MeOH/MeCN (50/50 v/v) + 0.1% FA. The binary gradient was set up as follows: for the first 0.5 minutes, eluent B was kept at 5%, from 0.5 to 3 minutes eluent B was increased to 95% and kept at 95% from 4 to 6 minutes, then eluent B was dropped to a constant 5% from 6 to 7 minutes. The tabulated binary gradient is illustrated in **Supplementary Table 1**. The equilibration time was 1 minute, and the flow rate was kept at 0.5000 mL/min throughout the runs. During all experiments the column oven temperature was maintained at 40°C using the column thermostat, and the autosampler at 15°C. All measurements were done in multiple reaction monitoring (MRM) mode. The conditions of the ion source were as follows: source temperature 450°C, curtain gas 30 psi, collision gas high, ion source gasses (sheath and drying gas) 80 psi. The LC-MS/MS Instrument was controlled using the Analyst 1.7.0 software and data acquisitions were processed using MultiQuant™ 3.0.2 Software.

### 2.6 Method validation

Linearity, limits of detection (LODs), limits of quantification (LOQs), matrix effects, selectivity, precision (repeatability and reproducibility within-laboratory), recovery, and decision limit (CCα) were determined. Calibration curves were constructed from blank samples that were spiked to five concentrations which covered the analyte concentration range expected to be present in the experiments, **Table 2**. Matrix-matched calibration curves are used because the matrices in this study were complex; their components interfered with the analyte and altered the instrument response towards the analyte of interest. Peak area vs. mycotoxin concentrations were plotted to generate calibration curves. Linearity was evaluated visually and statistically using the product-moment correlation coefficient, R^2^.

**Table 2:** Detailed dilution scheme for preparation of calibration standards.

Working standard solution		1	2	3	4	5
**Volume of mixed stock solution added to 1 mL of sample extract**		0	25	100	150	200
**Concentration of each mycotoxin (µg/Kg)**	AFB_1_, B_2_, G_1_ & G_2_	0	2.5	10	15	20
	FB_1_, FB_2_ & T2-Toxin	0	62.5	250	375	500
	HT2-Toxin & ZEA	0	25	100	150	200
	OTA	0	10	40	60	80

LOD is the lowest concentration of an analyte that can be detected but is not quantifiable under the stated conditions of the method. On the other hand, LOQ is described as the minimum concentration of an analyte that is quantifiable. Since linear regression was used to linearly model the relationship between the concentration (x) of mycotoxins and the response (y), it can be expressed in a model such as y=bx +c where; y is the response signal, x is the concentration, b is the slope of the curve, and c is y-intercept of the curve. This model can then be used to compute the LOD and LOQ of the analytical method, which can be calculated using Equations 1 and 2. Where S_a_ is the standard deviation of the response. In this study, the standard deviation was estimated using the y-residuals (ŷ) of the response.


Equ. 1 $$LOD=3\;\times \;\frac{S_{a}}{b}$$

Equ. 2 $$LOQ=10\;\times \;\frac{S_{a}}{b}$$

To evaluate recovery, 18 blank matrix samples were used. Six aliquots were fortified at each of 0.25, 1, and 1.5 times the maximum residue limit (MRL) of each mycotoxin. Samples were then extracted, and their concentrations were determined. The recovery for each sample and coefficients of variation (CVs) from the six results of each level were calculated using Equations 3, 4, and 5, respectively, where $\bar{x}$ is the mean concentration.


Equ. 3 $$\%Recovery=100\;\times \;\frac{measured\;content}{fortification\;level}\;$$

Equ. 4 $$SD=\sqrt{\sum^{}\frac{{\left(x_{i}-\bar{x}\right)}^{2}}{\left(n-1\right)}}$$

Equ. 5 $$Repeatability\;CV=\frac{SD}{\bar{x}}\;\times 100\%\;$$

The precision of this analytical method was determined based on quality control (QC) samples. Six replicate QC samples fortified with the multi-mycotoxin standard to yield concentrations equivalent to 0.25, 1, and 1.5 times the MRL of each mycotoxin were prepared and analyzed (n = 6 for 3 different concentrations). These steps were repeated in consecutive weeks to evaluate the within-laboratory repeatability. The mean concentrations, SD (Equation 4), and CV_r_ (%) (Equation 5) of the fortified samples were calculated. The total ion flow chart and MS/MS spectrum of each mycotoxin for the QC sample fortified with multi-mycotoxin standard at MRL are presented in **Supplementary Figure 1** and **Supplementary Figure 2** respectively.

“CCα is the limit at and above which it can be concluded with an error probability of α that a sample is non-compliant” (EU, 2021). CCα was determined by analyzing 20 blank samples per matrix fortified with the analyte (s) at MRL concentrations. The mean concentration at MRL plus 1,64 times the corresponding standard measurement uncertainty at MRL equals CCα (α = 5%) (Equation 6). CCα values for each mycotoxin are shown in **Table 3**.

**Table 3:** Percentage Recoveries, coefficients of variation and decision limits from the validation study.

Mycotoxin	Recovered concentration (µg/Kg)			% Recovery			Repeatability CV (%)			Reproducibility CV (%)			CCα
	0.25 MRL	1.0 MRL	1.5 MRL	0.25 MRL	1.0 MRL	1.5 MRL	0.25 MRL	1.0 MRL	1.5 MRL	0.25 MRL	1.0 MRL	1.5 MRL	
**AF-B_1_**	2.47	9.01	14.41	98.69	90.10	96.06	7.51	11.86	5.30	11.23	11.86	1.82	11.46
**AF-B_2_**	2.40	9.21	15.27	96.09	92.07	101.79	12.04	8.39	13.37	14.87	1.76	7.58	12.10
**AF-G_1_**	2.43	9.24	14.84	97.29	92.44	98.94	10.63	9.87	10.59	9.20	9.87	3.51	12.43
**AF-G_2_**	2.28	8.91	14.54	91.29	89.11	96.91	10.39	10.20	12.77	10.39	10.20	12.77	12.64
**FB_1_**	54.02	224.00	324.94	86.93	89.60	86.92	13.09	14.11	12.03	13.12	14.11	1.65	247.13
**FB_2_**	50.48	229.96	327.84	80.77	91.99	87.42	8.12	9.60	8.27	8.92	9.60	6.80	245.71
**HT2**	22.57	91.33	141.21	90.29	91.33	94.14	7.96	11.88	10.25	3.12	5.10	10.25	99.70
**T2**	67.93	244.21	365.13	108.68	97.69	97.31	7.79	8.59	6.20	5.14	3.15	5.17	249.37
**OTA**	10.98	39.66	59.28	109.83	99.16	98.80	10.46	7.73	5.25	9.23	4.02	2.45	46.25
**ZEA**	22.23	93.74	145.91	88.91	93.74	97.27	13.59	10.67	8.11	7.92	10.67	3.10	111.24


Equ. 6 CCα=MRL+(1.64 × standard deviation at MRL)

## 3 Results and discussion

### 3.1 LC-MS/MS optimization

ESI and MS/MS parameters were carefully optimized to obtain the ideal analytical conditions for detection of the selected mycotoxins (**Table 4**). Fragmentation of all target mycotoxins was studied through a full-scan analysis. The most intense ion in the fragmentation was selected for quantitation, and the second intense ion was chosen for qualification. According to the Commission Implementing Regulation (EU) 2021/808 of 22 March 2021, at least two ion transitions should be used for the monitoring of each target analyte in an instrumental method (EU, 2021). Furthermore the literature reports most of the product ions that have been used for the quantification of multiple mycotoxins (Jo et al., 2021; Pantano et al., 2021; Salim et al., 2021). MRM was established in a positive ion mode. Complete MS/MS parameters are summarized in **Table 4** and **Supplementary Figure 3** shows chromatograms of each mycotoxin.

**Table 4:** MS/MS parameters for all analytes, including retention time (min), ionization, precursor ion (m/z), product ion (m/z) and collision energy (V).

Mycotoxin	Retention time (min)	Ionization	Precursor ion (m/z)	Product ion (m/z)	Collision energy (V)
**Aflatoxin B_1_**	3.15	[M+H] ^+^	313.0	285.0 213.0	30 24
**Aflatoxin B_2_**	3.09	[M+H] ^+^	315.0	287.0 259.0	34 30
**Aflatoxin G_1_**	3.03	[M+H] ^+^	329.0	243.0 213.0	30 20
**Aflatoxin G_2_**	2.96	[M+H] ^+^	331.0	245.0 189.0	30 22
**Fumonisin B_1_**	2.99	[M+H] ^+^	722.0	334.0 352.0	26 22
**Fumonisin B_2_**	3.23	[M+H] ^+^	706.0	336.0 318.1	18 16
**Ochratoxin A**	3.62	[M+H] ^+^	404.0	358.0 239.0	12 12
**HT2-Toxin**	3.34	[M+H] ^+^	447.0	345.0 285.0	18 14
**T2-Toxin**	3.54	[M+H] ^+^	489.0	327.0 387.0	22 16
**Zearalenone**	3.66	[M+H] ^+^	319.1	185.0 187.0	10 10

### 3.2 Optimization of extraction procedure

#### 3.2.1 Effect of the type of extraction solvent

**Figure 1:**
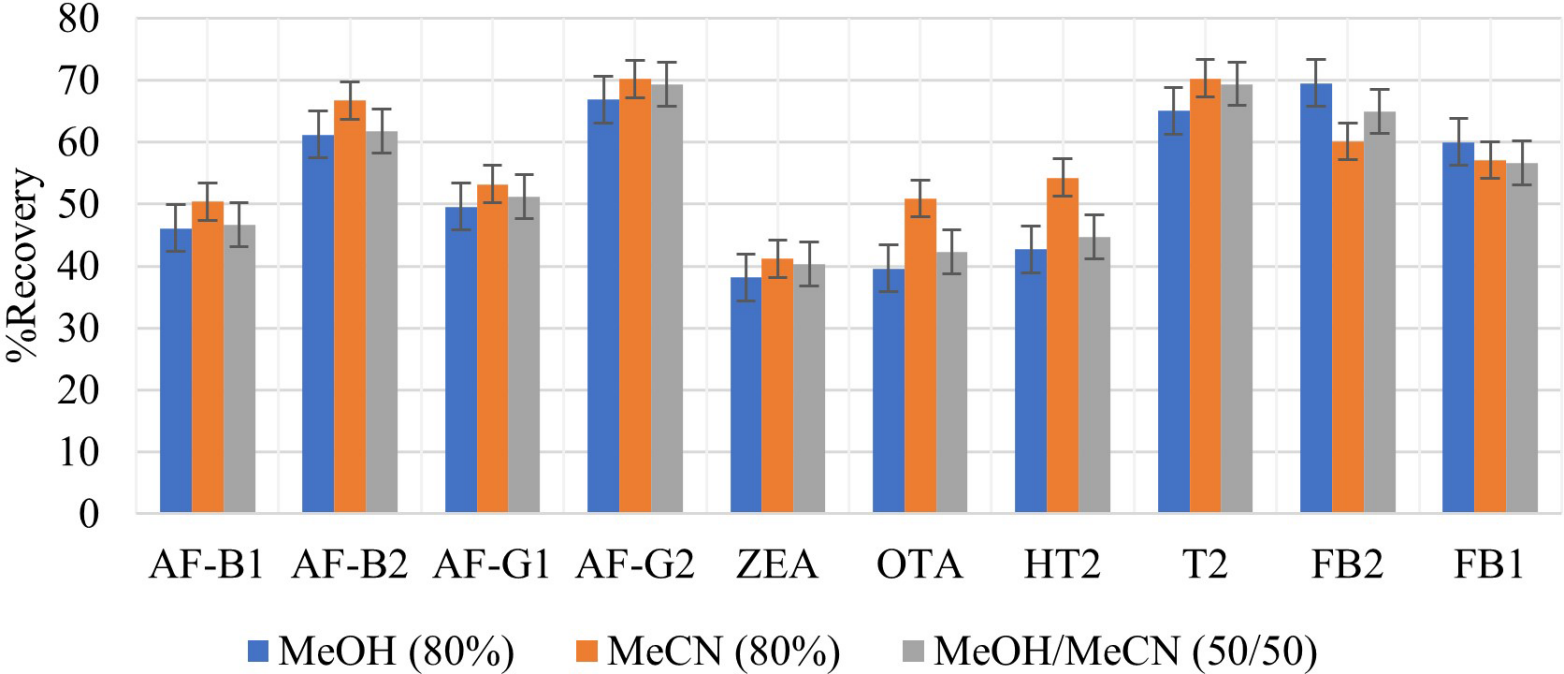
Effect of different extraction solvents, 80% MeOH, 80% MeCN and 80% MeOH/MeCN (50/50 v/v) on extraction recovery of each mycotoxin.

The use of 100% organic solvents to extract multiple mycotoxins has been shown to be ineffective in extracting all analytes, especially the polar mycotoxins such as trichothecenes (HT2 and T2-toxin) (Nakhjavan et al., 2020). MeCN, MeOH, and H_2_O have been used as mycotoxin extraction solvents. H_2_O improves the extraction of polar mycotoxins. In this study, three solvent mixtures were investigated for their suitability as extraction solvents for selected mycotoxins; 80% MeOH, 80% MeCN, and 80% MeOH/MeCN (50:50 v/v). **Figure 1** shows the analyte recoveries from using the selected extraction solvents. Extraction with 80% MeCN yielded the highest recoveries for all analytes except the FBs; therefore, it was selected as the extraction solvent to be used in this work. FBs are highly polar compounds, and that explains why higher recoveries were observed when extracting with 80% MeOH. Among all analytes, ZEA had the lowest recovery (41.2%) because it is non-polar, and 80% MeCN is polar. These findings are similar to those made by Pantano et al. (2021) who recorded 65.0% recovery for ZEA, which was among the lowest recoveries in their study. Unlike MeOH, MeCN reduces the extraction of matrix components and is able to extract analytes that have different polarities (González-Curbelo et al., 2015). This explains the better recoveries achieved using 80% MeCN since only the substances of interest were extracted from the matrices, leading to few interferences. All mycotoxins analyzed in this study are soluble in MeCN.

#### 3.2.2 Effect of formic acid on extraction solvent’s efficiency

**Figure 2:**
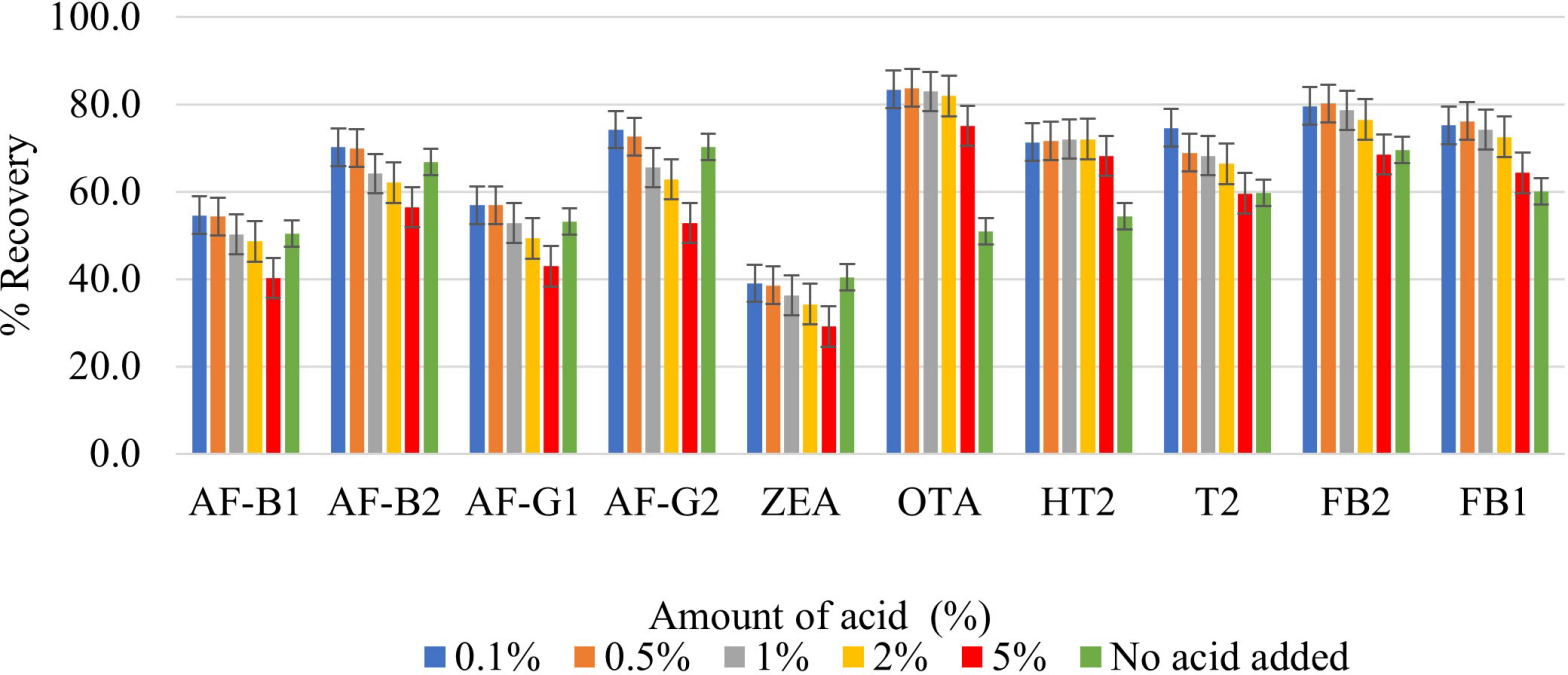
Effect of 0.1%, 0.5%, 1%, 2%, 5% formic acid on the extraction recovery of each mycotoxin.

The addition of an organic acid to the extraction solvent is necessary to improve the extraction of mycotoxins that have a carboxylic acid moiety (FB_1_, FB_2_ OTA, and T2-toxin), because these mycotoxins are more soluble in acidic solvents. While some studies have used acetic acid for this purpose (Jettanajit and Nhujak, 2016; González-Jartín et al., 2021), most report the use of FA, possibly because FA is a much simpler organic acid. FA in various percentages has been used by different researchers to decrease the pH of the extraction solvent and effectively extract multiple mycotoxins (Beltrán et al., 2009; Hosseini Asl et al., 2019; Jo et al., 2021; Pantano et al., 2021). In this study, different percentages of FA were added to 80% MeCN to optimize the extraction of FB_1_, FB_2_ OTA, and T2-toxin. **Figure 2** illustrates the percentage recoveries of each analyte when using various FA percentages in the extraction solvent (0.1, 0.5, 1, 2, and 5%). The addition of 0.1% FA resulted in the highest analyte recoveries; as an illustrative example, the recoveries of FB_1_, FB_2_, OTA, and T2-toxin in maize increased from 69.5%, 60.0%, 50.9%, and 59.7% (without FA) to 75.2%, 79.6%, 83.4%, and 74.6%, respectively (**Figure 2**). Although Beltrán et al. (2009) also used 0.1% FA to extract multiple mycotoxins in maize kernels, they did not report any optimization that influenced their choice of 0.1% FA. Other studies have reported the use of relatively larger amounts of FA the for extraction of multiple mycotoxins in cereals, such as 20% FA in MeCN (Pantano et al., 2021) and 10% FA in H_2_O (Jo et al., 2021). It is crucial to develop techniques that use FA at relatively low levels because its usage in high quantities has the potential to be hazardous to the environment.

#### 3.2.3 Effect of agitation time

**Figure 3:**
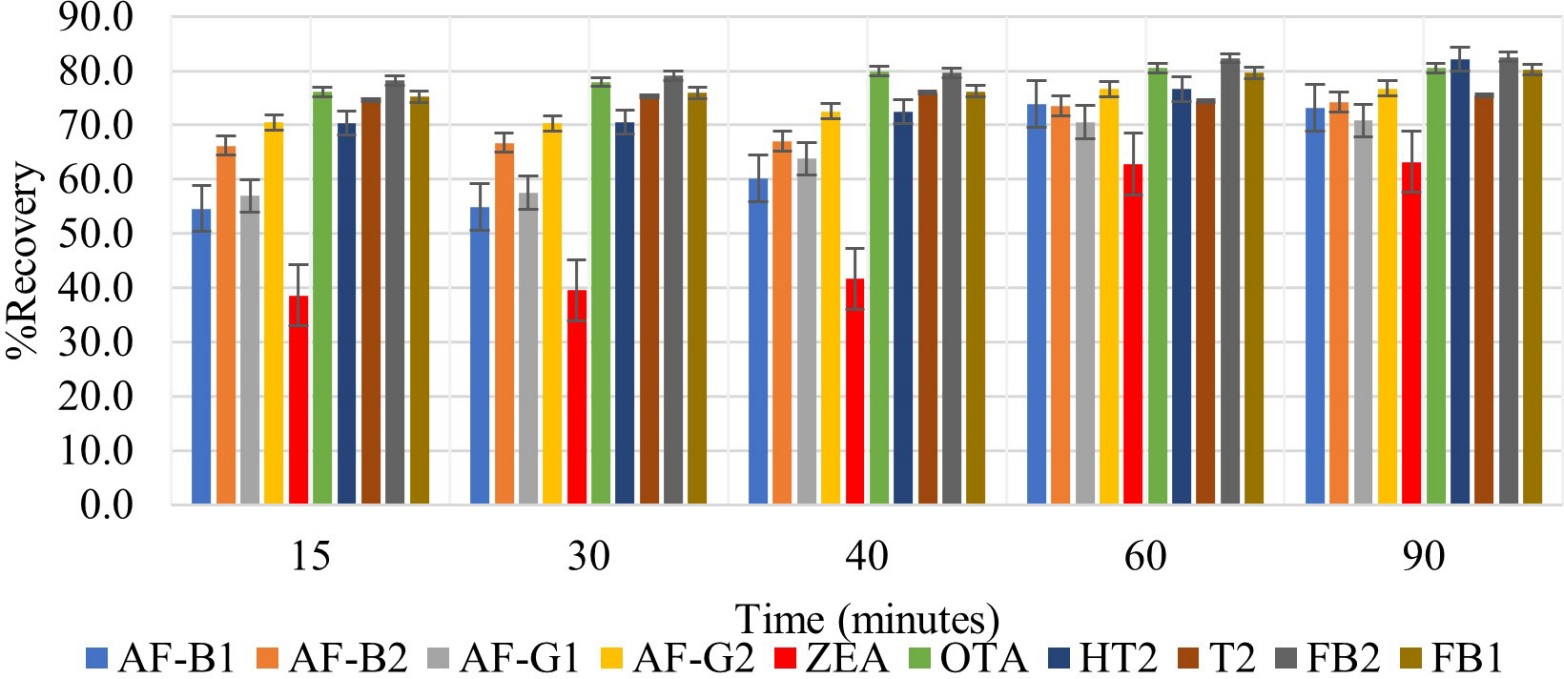
Effect of 15-, 30-, 40-, 60- and 90-minutes agitation time on the extraction recovery of each mycotoxin.


The mycotoxins analyzed in this study interact differently with the extraction solvent due to their various polarities. It is therefore important to optimize agitation time in order to allow maximum interaction of the extraction solvent with the matrix to effect maximum extraction of all analytes. None of the previously reported QuEChERS methods for determination of multiple mycotoxins in cereals focused on optimization of this factor (Beltrán et al., 2009; Arne and Mehdi, 2014; Jettanajit and Nhujak, 2016; González-Jartín et al., 2021; Jo et al., 2021; Pantano et al., 2021). It should be noted that time spent agitating samples after the addition of the extraction solvent influences the extraction efficiency and the peak intensities of analytes. The effect of different agitation times (15, 30, 40, 60, and 90 minutes) on analyte recovery was assessed. Increasing agitation time increased the recovery of all analytes (**Figure 3**). Very high analyte recoveries were observed after agitating for 60 and 90 minutes, the most notable was ZEA, which increased from 38.6% at 15 minutes to 62.8% and 63.2% at 60 and 90 minutes, respectively. This could be due to its non-polar and hydrophobic nature. This suggests that ZEA will thus require more time to be effectively absorbed by the relatively polar extraction solvent. As expected, the highest recoveries were among mycotoxins that have an acid moiety (FB_1_, FB_2_ OTA, and T2-toxin), and this could be because of their high affinity with the extraction solvent. A paired t-test was used to find out whether the difference between recoveries observed after agitating for 60 and 90 minutes was significant (**Equation 7**).

Equ.7 $$t_{statistic}=\;\left|\bar{d}\right|\;\frac{\sqrt{n}}{S_{d}}$$

Where d is the difference between the pair of results, n is the number of paired results, and S_d_ is the standard deviation d. The 60 minutes shaking time was selected for this study because, according to statistical analysis, |t_statistic_| was less than t_critical_ (1.51< 2.26) and this meant that there was no significant difference between the recoveries observed after shaking for 60 and 90 minutes (**Supplementary Table 2**). Thus, 60 minutes was selected for a faster extraction procedure.

#### 3.2.4 Effect of salt addition

**Figure 4:**
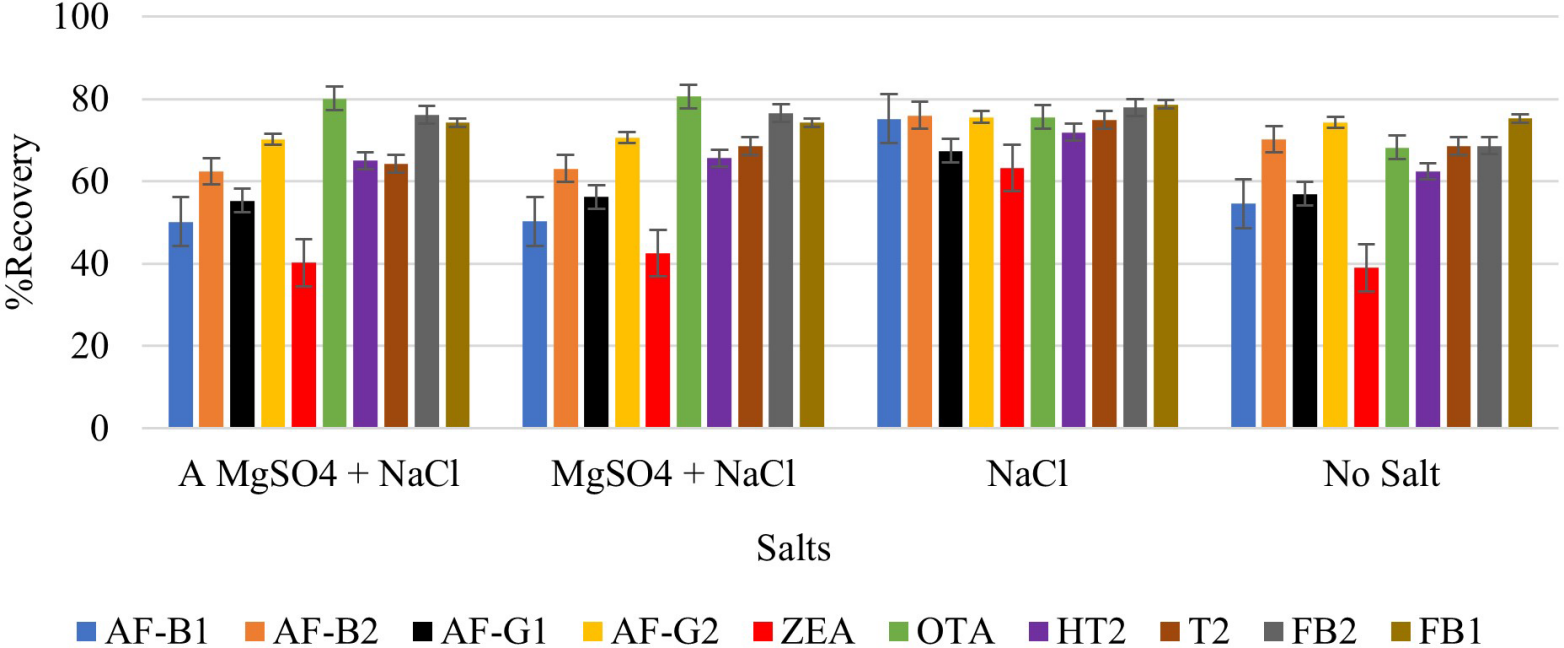
Effect of salt addition (NaCl and MgSO_4_) on the extraction recovery of each mycotoxin.

**Figure 5:**
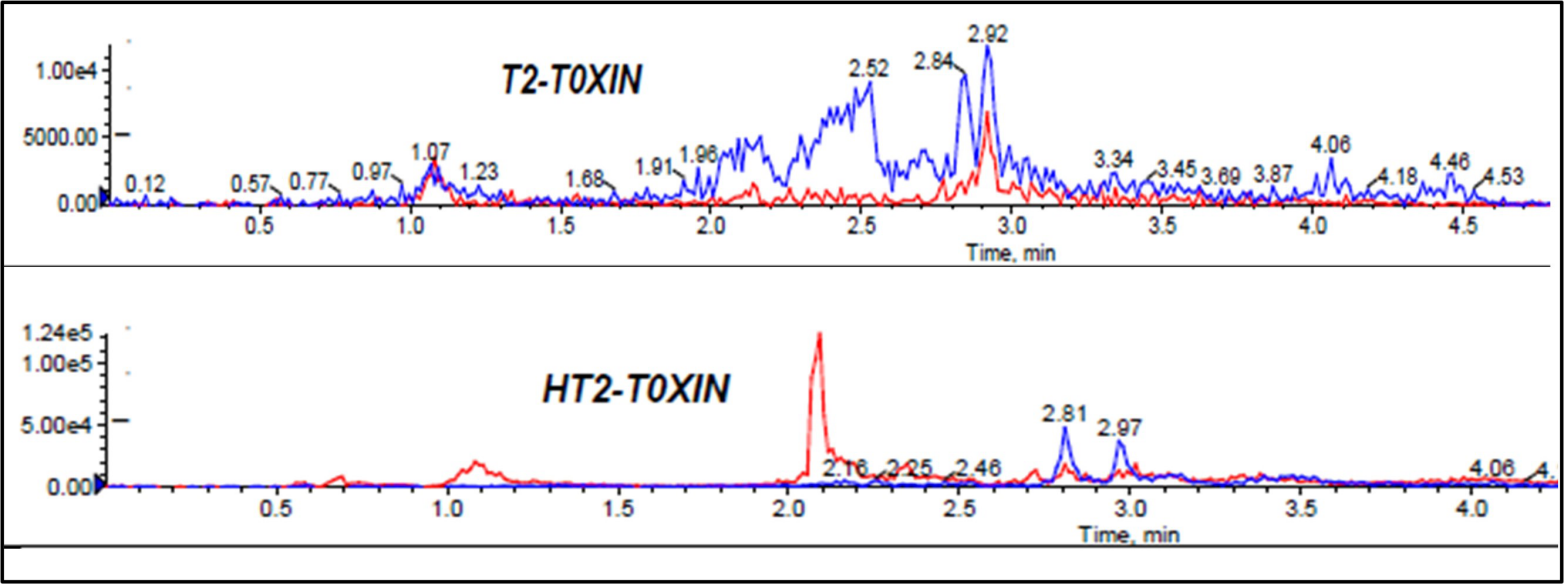
Effect of no salt addition to mycotoxin chromatograms, T2 and HT2 toxins chromatograms used as samples.

**Figure 6:**
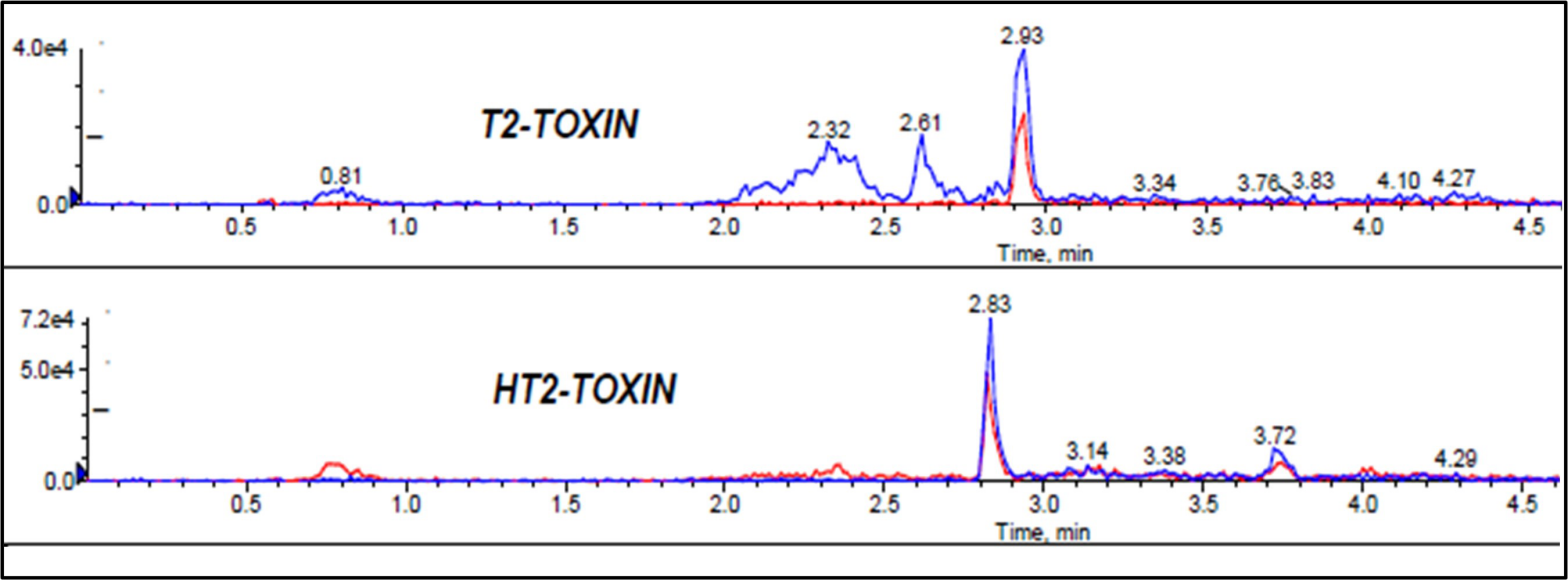
Effect of NaCl addition to mycotoxin chromatograms, T2 and HT2 toxins chromatograms used as samples.

Extraction of multi-component analytes is challenging mainly because of the different physicochemical properties of the analytes. It can also be difficult due to matrix effects and interferences. Salting in QuEChERS is done to induce proper separation of the organic and aqueous phases (González-Curbelo et al., 2015) and reduce the amount of water in the final extract, leading to cleaner extracts and reduced emulsions. Anhydrous MgSO_4_ and hydrated MgSO_4_ are the commonly employed drying agents in the QuEChERS technique, while NaCl is used to induce phase separation (Anastassiades et al., 2003; González-Curbelo et al., 2015). The effect of salt addition prior to centrifuging was investigated under the following conditions: (i) 4g Anhydrous MgSO_4 + _1g NaCl, (ii) 4g MgSO_4 + _1g NaCl, (iii) 1g NaCl, and (iv) No salt. Higher analyte recovery (**Figure 4**) and chromatograms with less noise were observed for all analytes after the addition of NaCl only. **Figures 5**, **6** are sample presentations of the chromatograms without and with NaCl addition using T2 and HT2 toxin. Upon visual inspection, the extracts from which only NaCl was added were much cleaner than the rest, meaning that NaCl reduces the extraction of interferants and other unwanted matrix components, hence resulting in less noise recorded in the chromatograms. NaCl is also said to provide a clear and distinct phase that facilitates the penetration of mycotoxins into the organic layer (Sirhan et al., 2014). These observations may also be influenced by the agitation time because the longer the matrix spends in the organic solvents, the more it disperses, which later makes it easier for NaCl to be used as an extract cleaner. The experiments also revealed that using anhydrous MgSO_4_ and ordinary MgSO_4_ yields significantly different results, as confirmed by a paired t-test where |t_statistc_| > t_critical_ (2.48> 2.26) (**Supplementary Table 3**). Due to its strong exothermic nature, anhydrous MgSO_4_ raises the temperature of the material inside the falcon tube. This explains the lower recoveries observed after adding it compared to the hydrated MgSO_4._

#### 3.2.5 Effect of extract dilution and concentration

**Figure 7:**
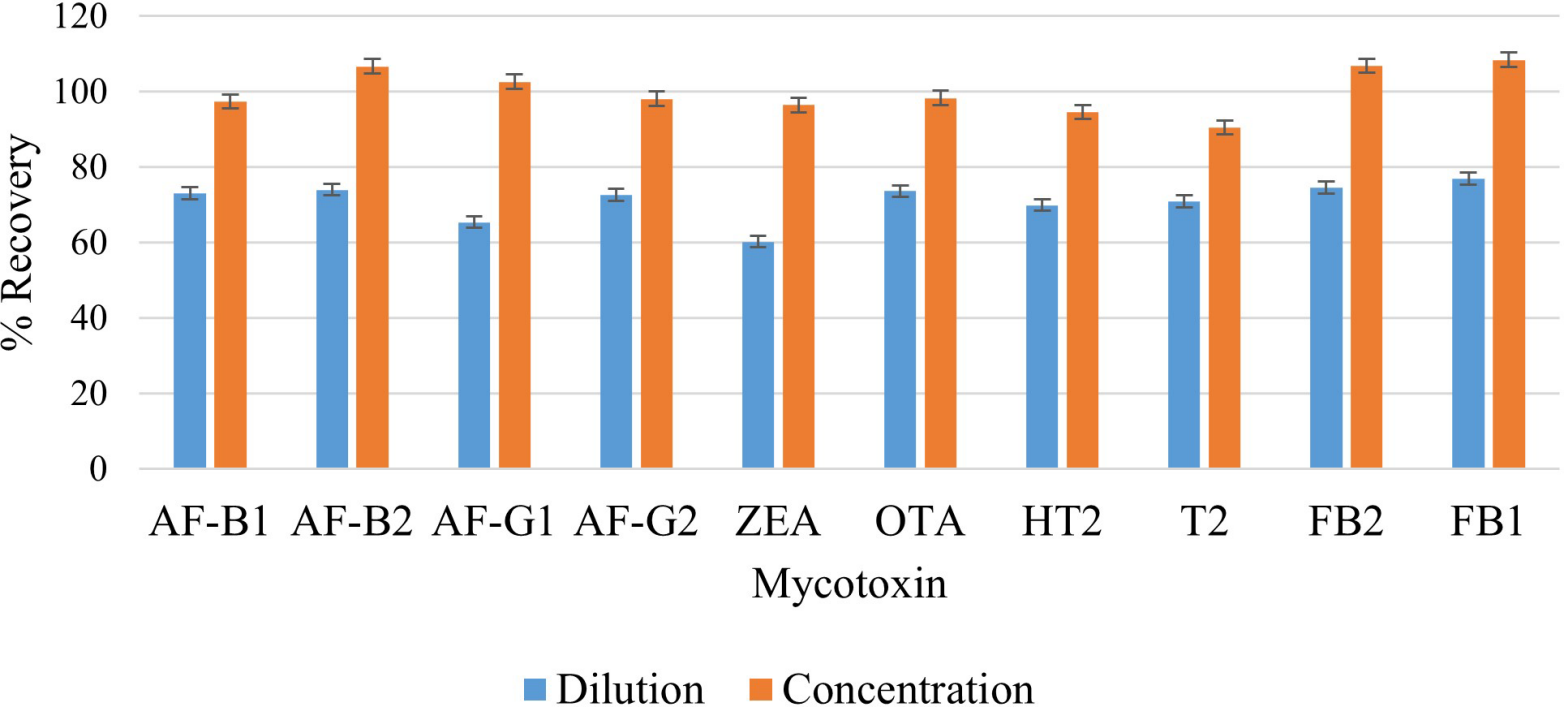
Effect of dilution and concentration on extraction recovery of each mycotoxin.

According to Sirhan et al. (2014), in order to facilitate the release of analytes from the matrix, it is necessary to dilute sample extracts. This minimizes matrix effects and reduces the number of competing molecules per drop area. However, dilution may also reduce the sensitivity and result in poor detection limits. On the other hand, concentrating analytes in the extracts may improve sensitivity and lead to better detection limits while suppressing other analytes in a multi-determination study. Previous studies do not report investigations of these factors. Therefore, these two factors need to be studied and optimized to find the most optimal treatment of the extract prior to injection into the LC-MS/MS. In this study, the effect of dilution and concentration were investigated by subjecting one extract to dilution, where 500 µL of the extract was mixed with 500 µL of the reconstitution solution (MeOH/H_2_O 50:50 v/v), then filtered and injected into LC-MS/MS. The second extract was collected (≈ 6 mL), concentrated under a nitrogen stream to 500 µL, reconstituted with 500 µL of MeOH/H_2_O (50:50 v/v), then filtered and injected into LC-MS/MS. Higher analyte recoveries were observed from the sample that was concentrated prior to injection into the LC-MS/MS (**Figure 7**). The |t_statistic_| was greater than t_critical_ (9.66 > 2.26), meaning that the difference is significant (**Supplementary Table 4**). Therefore, the concentration step was included in the extraction procedure because it improves instrument sensitivity to the selected mycotoxins. Recoveries ranging from 90.5% to 108.4% were observed, and these are much higher compared to those recorded from studies that diluted the extract prior to injection into LC-MS/MS. Jo et al. (2021) diluted 400 µL of their supernatant with 100 µL H_2_O and 100 µL MeCN, and their recoveries ranged from 70.1% to 106.3% for cereal based feedstuffs. Similarly, Arne and Mehdi (2014) reported recoveries ranging from 84.24% to 105.06% for rice samples after diluting 500 µL of the supernatant extract with 500 µL of H_2_O.

### 3.3 Method validation

#### 3.3.1 Calibration function, LODs and LOQs

Calibration curves for each analyte are presented in **Supplementary Figures 4**-**12**. The R^2^ value, were greater than 0.98 in all cases, suggesting that the recorded signals can be well correlated with the actual concentration of the mycotoxins. Furthermore, one can directly predict the concentration of the analytes from the curve over the concentration ranges used in these experiments. The linearity ranges, LODs, and LOQs for the selected mycotoxins are listed in **Table 5**.

**Table 5:** Linearity ranges, limits of detection (LODs), limits of quantification (LOQs) and matrix effects for the selected mycotoxins.

Mycotoxin	Linearity Range (µg/Kg)	LOD (µg/Kg)	LOQ (µg/Kg)	R^2^	SSE%		
					Maize	Sorghum	
**AFB_1_**	2.5 – 20	0.36	1.19	0.9997	81.54	76.32
**AFB_2_**	2.5 – 20	0.53	1.76	0.9994	74.35	77.21
**AFG_1_**	2.5 – 20	0.57	1.90	0.9994	74.89	65.16
**AFG_2_**	2.5 – 20	0.16	0.53	0.9999	78.09	67.65
**FB_1_**	62.5 – 500	14.15	47.17	0.9994	101.23	76.86
**FB_2_**	62.5 – 500	12.99	43.30	0.9964	102.41	80.94
**T2**	62.5 – 500	26.78	89.28	0.9979	69.41	87.20
**HT2**	25 – 200	8.47	28.23	0.9998	36.29	51.72
**OTA**	10 – 80	1.47	4.89	0.9998	78.52	89.90
**ZEA**	25 – 200	7.73	25.76	0.9989	36.75	41.63

#### 3.3.2 Matrix effects

The signal suppression/enhancement (SSE) for all analytes in each matrix was calculated using Equation 8 to assess any ME brought on by biological components of the maize and sorghum samples. Slope comparisons were made between solvent and matrix-matched standards to evaluate MEs. A 100% SSE value indicates that the matrix has no discernible impact on the strength of the MS signal. For signal suppression, the difference between these slopes is less than 100%, while it is more than 100% for signal enhancement.


Equ.8 $$Matrix\;effect\;\left(\%\right)=100\;\times \;\frac{Slope_{matrix-matched\;standard\;calibration}}{Slope_{solvet\;standard\;calibration}}$$

**Table 5** Shows data on the calculated SSE for selected mycotoxins in maize and sorghum. The data shows signal enhancement of FB_1_ and FB_2_ in maize by 1.23% and 2.41%, respectively. Ion suppression for all other mycotoxins varied in maize and sorghum. From these results, it was determined that the ion SSE depends on both the analyte and matrix together.

#### 3.3.3 Recovery and precision

Satisfactory recoveries were obtained for all mycotoxins. Recoveries ranged from 80.77% to 109.83% for all mycotoxins. These were within the recommended range of 70% to 120% by Commission Implementing Regulation (EU) 2021/808 of 22 March 2021 (EU, 2021). Satisfactory results for the within-laboratory reproducibility of the method were observed, with CV values typically below 15% with very few exceptions, **Table 3**. According to Regulation (EC) no. 401/2006 the within laboratory repeatability should be as low as possible (Regulation, 2006). The data in **Table 3** also shows CV values below 15%, which is considered low enough and indicates good precision.

#### 3.3.4 Decision limit (CCα)

CCα is a concentration at and above which, with an error probability of 1 – α, a decision can be made that if a signal is detected, it is not noise and the analyte detected is truly present or above the MRL. The CCα values were calculated according to Equation 6 and are listed in **Table 3**. They are close to the MRL values for each mycotoxin, which means the method can detect and quantify mycotoxins at the permit limits accurately.

### 3.4 Application to maize and sorghum samples

Concentrations of the selected mycotoxins in maize and sorghum samples are show in **Supplementary Table 5**. The most frequently detected mycotoxins were AFB_1_, AFB_2_, AFG_1_, AFG_2_, FB_1_, FB_2_, and ZEA, with detections in 5, 4, 3, 3, 5, 5, and 3 samples, respectively. In addition, T2 and HT2 toxins were detected in one of the 20 samples. Ten samples had detectable mycotoxins, and three showed 4 or more mycotoxins. Maize samples were the most contaminated with AFs, FBs, and ZEA. FB_1_ and ZEA were simultaneously detected indicating that they could be co-occurring toxins in Botswana food stuffs. Therefore. AFs, FBs and ZEA appear to be the more important contaminants in maize and sorghum in Botswana.

Amounts of these toxins were detected in the range of 1.27 - 4.07 µg/Kg for AFB_1_, 1.045 - 2.175 µg/Kg for AFB_2_, 1.30 - 2.835 µg/Kg for AFG_1_, 1.335 - 2.11 µg/Kg for AFG_2_, 18.345 - 25.64 µg/Kg for FB_1_, 11.86 - 18.32 µg/Kg for FB_2_, and 13.73 - 20.625 µg/Kg for ZEA. All detected concentrations except for AFB_1_ were below the maximum permitted levels by the EU. Two samples of maize had levels of AFB_1_ (2.545 and 4.07 µg/Kg) above the maximum permitted levels of 2 µg/Kg in cereals intended for human consumption (EU, 2021). Thus indicating exposure to high levels of AFB_1_. These findings reflected the possible contamination of Botswana’s maize and sorghum by mycotoxins, especially, maize which was also reported by Masitha, Sereme-Mothobole and Kabelo (Masitha et al., 2019), who identified mycotoxigenic fungi such as *Aspergillus (flavus, niger), Fusarium (proliferatum, fujikuroi)*, and *Alternaria* in maize and sorghum samples from Botswana. These results suggest that our method can be applied in the screening of mycotoxins in cereals such as maize and sorghum.

## 4 Conclusions

A quick, economical, and environmentally friendly analytical method based on QuEChERS-LC-MS/MS was developed and validated for the simultaneous determination of ten mycotoxins in maize and sorghum grains. Extraction solvent, agitation time, salt addition, dilution, and concentration of supernatants in the extraction procedure were all optimized by the OFAT approach. This study revealed that optimization of the QuEChERS extraction procedure for different matrices is crucial, as the different stages of this technique affect analyte recovery in different ways. The method was validated with respect to linearity (R2 > 0.98), limits of detection (0.16 to 26.78 µg/Kg), limits of quantification (0.53 to 89.28 µg/Kg) matrix effects, selectivity, precision (repeatability and reproducibility within-laboratory), percentage recoveries (80.77% to 109.83%) and decision limits. The developed method was applied to 20 real samples (10 maize and 10 sorghum). Half of the samples had detectable mycotoxins (AFs, FBs, T2, HT2, and ZEA). The most frequently occurring were the AFs, and two maize samples had levels of AFB1 above the maximum permitted level of 2 µg/Kg (2.55, 4.07 µg/Kg). Thus indicating the possibility of exposure for Botswana to high levels of mycotoxins, especially the most toxic of them all, AFB1. It is recommended that statistical tools such as response surface methodology be used to further optimize these factors in the next studies.

## Data availability statement

The original contributions presented in the study are included in the article/**Supplementary Material**. Further inquiries can be directed to the corresponding author.

## Author contributions

MM collected samples, performed the experiments, analyzed the data, and wrote the manuscript with guidance from TR and DM. TR assisted with design of experiments, method validation and execution of experiments. DM coordinated the project. IC assisted with coordination of the project and data analysis. All authors contributed to the article and approved the submitted version.
