# Supplementary material for: Development and validation of a QuEChERS-LC-MS/MS method for determination of multiple mycotoxins in maize and sorghum from Botswana
Source: Front Fungal Biol. 2023 Aug 3;4:1141427. doi: 10.3389/ffunb.2023.1141427 (PMC10512389; doi:10.3389/ffunb.2023.1141427)
Supplement: Supplementary file 1 [file DataSheet_1.docx]

Supplementary Material

Development and validation of a QuEChERS-LC-MS/MS method for determination of multiple mycotoxins in maize and sorghum from Botswana

Mesha Mbisana^1*^, Tshepho Rebagamang^2^, Dikabo Mogopodi^1^, Inonge Chibua^1^

^1^Laboratory of Analytical Chemistry, Faculty of Science, Department of Chemistry, University of Botswana, Gaborone, Botswana

^2^Residues, Botswana National Veterinary Laboratory, Gaborone, Botswana

*** Correspondence:**Mesha Mbisana
[mimimbisana@gmail.com](mailto:mimimbisana@gmail.com) /

[Mbisanam@ub.ac.bw](mailto:Mbisanam@ub.ac.bw)

# Supplementary Tables

**Supplementary Table 1**. Binary gradient elusion profile for LC-MS/MS analysis

| Time (minutes) | Flow rate (mL/min) | Mobile phase A (%) | Mobile phase B (%) |
| --- | --- | --- | --- |
| 0.00 | 0.5000 | 95.0 | 5.0 |
| 0.50 | 0.5000 | 95.0 | 5.0 |
| 3.00 | 0.5000 | 5.0 | 95.0 |
| 4.00 | 0.5000 | 5.0 | 95.0 |
| 6.00 | 0.5000 | 95.0 | 5.0 |
| 7.00 | 0.5000 | 95.0 | 5.0 |

**Supplementary Table 2**. Paired t-test: assessment of significant difference between agitation times 60 and 90 minutes.

| t-Test: Paired Two Sample for Means | |  |
| --- | --- | --- |
|  |  |  |
|  | *60* | *90* |
| Mean | 75.07 | 75.89 |
| Variance | 31.409 | 35.54766667 |
| Observations | 10 | 10 |
| Pearson Correlation | 0.958062343 |  |
| Hypothesized Mean Difference | 0 |  |
| df | 9 |  |
| t Stat | -1.514714002 |  |
| P(T<=t) one-tail | 0.082072692 |  |
| t Critical one-tail | 1.833112933 |  |
| P(T<=t) two-tail | 0.164145384 |  |
| t Critical two-tail | 2.262157163 |  |

**Supplementary Table 3.** Paired t-test: assessment of whether Anhydrous MgSO_4_ and hydrated MgSO_4_ produce significantly different results.

| t-Test: Paired Two Sample for Means | |  |
| --- | --- | --- |
|  |  |  |
|  | *A MgSO4 + NaCl* | *MgSO4 + NaCl* |
| Mean | 63.78 | 64.81 |
| Variance | 153.6173333 | 145.2498889 |
| Observations | 10 | 10 |
| Pearson Correlation | 0.994627021 |  |
| Hypothesized Mean Difference | 0 |  |
| df | 9 |  |
| t Stat | -2.481867667 |  |
| P(T<=t) one-tail | 0.01744219 |  |
| t Critical one-tail | 1.833112933 |  |
| P(T<=t) two-tail | 0.03488438 |  |
| t Critical two-tail | 2.262157163 |  |
|  |  |  |

**Supplementary Table 4.** Paired t-test: assessment of whether there is significance difference between dilution and concentration.

| t-Test: Paired Two Sample for Means | |  |
| --- | --- | --- |
|  |  |  |
|  | *DILUTION* | *CONCENTRATION* |
| Mean | 71.12 | 82.61 |
| Variance | 24.39733333 | 13.37433333 |
| Observations | 10 | 10 |
| Pearson Correlation | 0.653538238 |  |
| Hypothesized Mean Difference | 0 |  |
| df | 9 |  |
| t Stat | -9.655462185 |  |
| P(T<=t) one-tail | 2.39356E-06 |  |
| t Critical one-tail | 1.833112933 |  |
| P(T<=t) two-tail | 4.78712E-06 |  |
| t Critical two-tail | 2.262157163 |  |

Supplementary Table 5. Measured concentrations of mycotoxins in maize and sorghum samples collected in Botswana

| Samples | Contents (Average ± standard deviation µg/Kg) (n=2) | | | | | | | | | |
| --- | --- | --- | --- | --- | --- | --- | --- | --- | --- | --- |
|  | AFB_1_ | AFB_2_ | AFG_1_ | AFG_2_ | FB_1_ | FB_2_ | T2 | HT2 | OTA | ZEA |
| Maize | - | - | - | - | 20.565±0.0778 | - | - | - | - | - |
|  | - | - | - | - | 18.345±0.4596 | 11.86±0.3536 | - | - | - | 13.73±0.2263 |
|  | 2.545±0.0071 | 2.03±0.0999 | 1.685±0.0778 | - | - | - | - | - | - | - |
|  | - | - | - | - | - | - | - | - | - | - |
|  | - | - | - | - | 15.32±0.0002 | - | - | - | - | - |
|  | - | - | - | - | - | - | - | - | - | - |
|  | 1.50±0.0701 | 1.75±0.0424 | 1.30±0.0999 | 1.335±0.0354 | - | - | - | - | - | 14.91±0.4243 |
|  | 4.07±0.7071 | 1.045±0.4953 | 2.835±0.0354 | 1.685±0.0495 | 25.64±0.7495 | - | - | - | - | - |
|  | - | - | - | - | - | 18.07±0.0450 | - | - | - | - |
|  | - | - | - | - | - | - |  | - | - | - |
| Sorghum | - | - | - | - | - | - | - | - | - | - |
|  | - | - | - | - | - | 14.67±0.5091 | - | - | - | - |
|  | - | - | - | - | - | 18.32±0.0882 | - | - | - | - |
|  | 1.97±0.1131 | 1.655±0.0495 | - | - | - | - | - | - | - | - |
|  | - | - | - | - | - | - | - | - | - | - |
|  | - | - | - | - | - | - | - | - | - | - |
|  | 1.27±0.0707 | 2.175±0.2192 | 1.615±0.0636 | 2.11±0.1414 | 20.94±0.3818 | 13.01±0.0002 | 31.625±0.5728 | 21.695±0.1061 | - | 20.625±0.5728 |
|  | - | - | - | - | - | - | - | - | - | - |
|  | - | - | - | - | - | - | - | - | - | - |
|  | - | - | - | - | - | - | - | - | - | - |

# Supplementary Figures

**Supplementary Figure 1.** Total ion flow chart for mycotoxins in the QC sample

**Supplementary Figure 2**. MS/MS spectra of all mycotoxins present in the QC sample

**Supplementary Figure 3.** MS/MS spectra of each mycotoxin in blank solvent

**Supplementary Figure 4.** AFB_1_ calibration curve

**Supplementary Figure 5.** AFB_2_ calibration curve

**Supplementary Figure 6.** AFG_1_ calibration curve

**Supplementary Figure 7.** AFG_2_ calibration curve

**Supplementary Figure 8.** FB_1_ calibration curve

**Supplementary Figure 9.** FB_2_ calibration curve

**Supplementary Figure 10.** HT2 calibration curve

**Supplementary Figure 11.** OTA calibration curve

**Supplementary Figure 11.** T2 calibration curve

**Supplementary Figure 12.** ZEA calibration curve
